# Transportation barriers, loneliness, and depressive/anxiety symptoms in older adults

**DOI:** 10.1093/geroni/igaf127

**Published:** 2025-11-12

**Authors:** Namkee G Choi, C Nathan Marti

**Affiliations:** School of Social Work, The University of Texas at Austin, Austin, Texas, United States; School of Social Work, The University of Texas at Austin, Austin, Texas, United States

**Keywords:** Depression, Anxiety, Driving cessation, Social engagement, Social support

## Abstract

**Background and Objectives:**

Transportation is crucial to maintaining social engagement in later life. Lack of transportation is significantly associated with depressive/anxiety symptoms in older adults. In this study, we examined the extent to which loneliness mediates the relationship between transportation barriers and depressive/anxiety symptoms.

**Research Design and Methods:**

Using the 2023 National Health and Aging Trends Study (*N *= 7,547; age 65+), we defined transportation barriers as transportation problems that kept older adults from visiting family/friends, attending religious services, attending club meetings, or going out for enjoyment. We fitted a path model, and to test the mediation effect, we used bootstrapped analysis to obtain estimates of the indirect effects and their 95% confidence intervals (CIs).

**Results:**

Of the study population, 4.4%, or 2 million people, reported transportation barriers. We found significant direct effects of transportation barriers on loneliness and depressive/anxiety symptoms and indirect effects of loneliness (0.40, 95% CIs = [0.23, 0.57], *z *= 4.57, *p *< .001) on depressive/anxiety symptoms. The ratio of the indirect effect to the total effect of transportation barriers on depressive/anxiety symptoms (indirect effect [0.40] + direct effect [0.50] = 0.90) was 0.44. The ratio of the indirect effect of loneliness to the direct effect of transportation barriers on depressive/anxiety symptoms was 0.80.

**Discussion and Implications:**

Loneliness significantly mediates the association between transportation barriers and depression/anxiety in older adults. Transportation should be recognized as both a mobility and a mental health issue. Policies to enable older adults to continue social engagement, decrease loneliness, and alleviate depression/anxiety are needed.

Innovation and Translational Significance: This study shows that transportation in later life is not just a mobility issue but a mental health determinant and that transportation services for continued social engagement should be included in broader strategies for reducing loneliness and depression/anxiety among older adults. Needed transportation policies include increased publicly funded transportation programs (e.g., through the Older Americans Act) and paratransit services more attuned to the needs and functional abilities of older adults; coordinated transportation services among senior centers, community organizations, and transportation providers for social, community, and recreational activities; and subsidies for expanding on-demand ride-hailing services for older adults.

Transportation is a fundamental component of older adults’ everyday functioning, enabling access to essential services and opportunities for social engagement and social interaction (Dickerson et al., 2019; Strogatz et al., 2020). Participation in social/community activities is especially vital for maintaining older adults’ physical, mental, and cognitive health and for fostering a healthy community, and transportation plays a central role in enabling such participation regardless of geographic context (Desai et al., 2023; Jones et al., 2023; Levasseur et al., 2020). Thus, transportation barriers, often resulting from the loss of driving privileges due to aging-related health problems (Ang et al., 2019; Maliheh et al., 2023; Wood et al., 2023), can significantly compromise physical, social, and psychological well-being. This is especially true in the car-centric context of the United States, where alternative transportation options are frequently limited or inaccessible for older adults. Non-driving older adults, particularly those who are socioeconomically ­disadvantaged and reside in areas lacking adequate public or community transportation, are less likely to leave their homes and more likely to experience reduced social engagement, increasing their risk for social isolation and loneliness (Dabelko-Schoeny et al., 2021; Lamanna et al., 2020; Williams et al., 2024).

While social isolation is objectively measured with one’s social network size, loneliness tends to be subjective and defined as “the unpleasant experience that occurs when a person’s network of social relationships is significantly deficient in either quality or quantity” (Perlman & Peplau, 1984, p. 15). Along with other life transitions such as bereavement and retirement, research has shown that loneliness in later life is commonly driven by disrupted social/community participation, particularly following driving cessation, and diminished social connectedness (Hajek et al., 2025; Tomida et al., 2024). Loneliness has been recognized as a major public health concern, associated with a range of adverse outcomes, including increased severity of depressive/anxiety symptom severity, dementia, cardiovascular disease, and premature mortality in older adults (Crowe et al., 2021; Holt-Lunstad et al., 2015; Müller et al., 2025; Oken et al., 2024; Roy et al., 2023; ­Valtorta et al., 2016).

Extensive research has also linked driving cessation and transportation barriers to increased depressive/anxiety symptoms among older adults, both cross-sectionally and longitudinally, even after accounting for sociodemographic, physical, and cognitive health statuses (Choi & DiNitto, 2016; Fenton et al., 2025; Missell-Gray & Simning, 2024). The elevated depressive/anxiety symptoms following driving cessation are likely mediated, at least in part, by loneliness resulting from diminished opportunities for social interaction and social engagement. Previous research has shown loneliness to be consistently linked to elevated risks of depression and anxiety (Cacioppo et al., 2006; Lee et al., 2021; Santini et al., 2020). Given the cascading effects of depressive/anxiety symptoms in later life on increased functional impairment, accelerated cognitive decline, reduced quality of life, higher health care utilization and costs, and premature mortality (Buczak-Stec et al., 2022; John et al., 2019; Wei et al., 2019), it is imperative to better understand the interrelationships among transportation barriers, loneliness, and depressive/anxiety symptoms in older adults. Despite its importance, the extent of the mediating role of loneliness in the relationship between transportation barriers and depressive/anxiety symptoms in later life remains underexplored.

## Study aims, theoretical framework, and hypotheses

**Figure 1.**
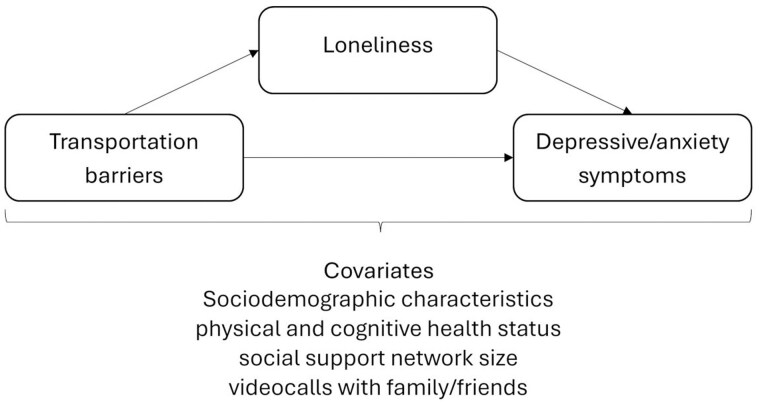
Graphic presentation of the mediation model.

In this study, using a nationally representative sample of community-dwelling Medicare beneficiaries ages 65 and older, we examined the direct effect of self-reported transportation barriers to participating in social/community activities on depressive/anxiety symptoms and the mediation effect of loneliness on the associations between transportation barriers and depressive/anxiety symptoms. The theoretical framework for the study is the social-ecological model that has guided previous studies of the challenges and opportunities associated with transportation availability, accessibility, affordability, acceptability, and adaptability (Dabelko-Schoeny et al., 2021; Jeghers et al., 2025) and social isolation and loneliness among older adults as a public health issue (Meehan et al., 2023). The social-ecological perspectives posit health outcomes to be shaped by the dynamic interplay of factors across individual, interpersonal, organizational, community, and societal levels (Webber et al., 2010). Specifically, transportation barriers are conceptualized as a community-level factor that can limit older adults’ participation in social/community activities, reducing opportunities for meaningful interpersonal interaction. This reduction in social engagement is likely to increase loneliness (an interpersonal- and individual-level factor), which in turn is associated with elevated depressive and anxiety symptoms at the individual level. The study hypotheses were (H1) depressive/anxiety symptoms would be positively associated with transportation barriers; (H2) loneliness would be positively associated with transportation barriers; and (H3) the association between transportation barriers and depressive/anxiety symptoms would be largely mediated by loneliness, controlling for sociodemographic characteristics, number of chronic illnesses, pain, cognitive health statuses, social support network size, and Internet-based virtual contact with family/friends. Figure 1 shows the graphical representation of the hypothesized relationships among these variables. The study findings will provide added insights into the mental health impact of transportation barriers on community-dwelling older adults and contribute to the ongoing discussion of innovative solutions to break the barriers.

## Method

### Data and sample

Data came from the 2023 National Health and Aging Trends Study (NHATS), which has been collecting data annually since its inception in 2011 from a nationally representative panel of Medicare beneficiaries (age 65+) regarding their physical, functional, cognitive, and sensory capacities; social, physical, and technological environments; and participation in valued activities. The NHATS sample has been replenished three times since its beginning, with the most recent one for the 2023 wave (https://www.nhats.org/). The 2023 NHATS data were collected in in-person interviews. In this study, we focused on 7,547 sample persons, representing 46.4 million older adults, who lived in their own homes or residential care communities (but not in nursing homes) and self-reported data (i.e., no proxy interview) in 2023. We excluded proxy-interviewed sample persons (*n *= 390) because the NHATS did not collect data on the sample persons’ loneliness (and other well-being indicators) from their proxy interviewees. This study, based on the analysis of de-identified public-use data, was exempt from the authors’ institutional review board review.

### Measures

#### Past-month automobile driving status and alternative transportation use

We constructed the past-month driving status variable (current driving vs not driving) from the respondent’s answers to the questions about driving “to get to places” in the last month, ceasing driving since the last interview (for continuing sample), and the last time the respondent drove. Those who drove last month were classified as current drivers, and those who stopped driving last year, stopped driving before last year, or never drove were classified as nondrivers. Respondents were also asked about the use of alternative transportation: (a) walking or using a wheelchair or scooter; (b) getting a ride from a family member, friend, or a paid helper; (c) using a van or shuttle service provided by the place of residence or for seniors or people with disabilities; (d) taking public transportation; and (e) taking a taxi, Uber, or Lyft; and (f) using any other means. We provided the alternative transportation use among non-drivers for descriptive purposes only.

#### Past-month transportation barriers

In NSDUH, sample persons’ self-reported transportation barriers, regardless of their driving status, were measured with questions about whether transportation problems ever kept them from doing each of the following activities in the preceding month: (a) visits with family/friends; (b) attendance in religious services; (c) participation in clubs, classes, or other organized activities than religious services (referred to as attendance in clubs/meetings hereafter); and (d) going out to dinner, a movie, to gamble, or to hear music or see a play (i.e., going out for enjoyment). We defined transportation barriers as an affirmative response to “transportation problem” for doing any of the four types of activities (yes = 1, no = 0). For descriptive purposes, we reported the number of these four activities that the sample persons participated in during the past month; whether they needed help going outside; and the past-month frequency of going outside the home (never/rarely [≤ once a week]; 2–4 times a week; 5+ times a week).

#### Depression/anxiety symptoms in the past month

In NHATS, depression/anxiety symptoms were assessed with the Patient Health Questionnaire-4 (PHQ-4) (Kroenke et al., 2009). The PHQ-4 includes the first two items (PHQ-2; had little interest or pleasure in doing things, and felt down, depressed, or hopeless) from the 9-item PHQ-9 for depression (Kroenke et al., 2003) and the first two items (GAD-2; felt nervous, anxious, or on edge, and have been unable to stop or control worrying) from the 7-item Generalized Anxiety Disorder Scale (Spitzer et al., 2006). Responses to each PHQ-4 item were based on a 4-point scale (0 = not at all; 1 = several days; 2 = more than half the days; 3 = nearly every day), with the total score ranging from 0 to 12. The unweighted Cronbach’s alpha for the PHQ-4 for the study sample was .77. The PHQ-4 scores were also used for descriptive purposes to categorize the symptom severity: no symptom (0–2), mild symptoms (3–5), and moderate/severe symptoms (6–12) (Kroenke et al., 2009).

#### Loneliness in the past month

This was assessed with a question, “During the last month, how often did you feel lonely?” The response categories were every day (=5), most days (5–6 days a week) (=4), some days (2-4 days a week) (=3), rarely (once a week or less) (=2), and never (=1). We treated it as a continuous variable, with the higher score representing greater loneliness.

#### Covariates

(1) Sociodemographic factors: These included age in 2023 (65–74 [reference category], 75–84, 85+); gender (female vs male [reference category]); race/ethnicity (non-Hispanic White [reference category], non-Hispanic Black, Hispanic, all other); marital status (married/partnered vs single [reference category]); and education (bachelor’s degree or higher vs no degree [reference category]). Household income and residence type (own home vs residential care facility) were reported for descriptive purposes only. (2) Health status indicators: These included the number of chronic medical conditions (0–8: arthritis, cancer, hypertension, heart disease, stroke, diabetes, lung disease, osteoporosis); activity-limiting chronic pain (yes or no); and dementia (no dementia, possible dementia, probable dementia, based on the most recently updated NHATS dementia classification algorithm [Kasper et al., 2024]); and (3) Social support indicators: These included social support network size, defined as the number of people (up to 5) the respondent talked to “about important things in life, including good or bad things that happened and problems or concerns;” and whether the sample person had any video calls with family and/or friends in the past month.

### Analysis

All analyses were conducted with Stata/MP 19.5’s svy function (College Station, TX) to account for NHATS’s stratified, multistage sampling design (Jiao & Freedman, 2024). First, we used *χ*^2^ and *t*-tests to compare those with and without transportation barriers with respect to sociodemographic and health characteristics, social support indicators, loneliness, and depressive/anxiety symptoms. Second, we fitted a path model for hypothesis testing (direct effect of transportation barriers and loneliness on depression/anxiety symptoms and the mediation effects of loneliness on the association between transportation barriers and depression/anxiety symptoms). Results for direct and mediation effects are reported as coefficients (B) and linearized standard errors (SE) with 95% confidence intervals (CIs). To test the statistical significance of loneliness as a mediator, we used bootstrapped (10,000 repetitions) analysis to obtain estimates of the indirect effect and its 95% CIs. We calculated the ratio of the indirect effect to the total effect (direct effect of transportation barriers on depressive/anxiety symptoms + indirect effect of the mediator). Note that, given the cross-sectional data, while we use terms such as direct and indirect effects from the mediation literature, we do not infer causal relationships, but the findings reflect associations. The cut-off for statistical significance was set at *p* < .05.

## Results

### Driving, social/community activity participation, and going-out frequency: comparison between those with and without transportation barriers

Table 1 shows that in 2023, 4.4% (95% CI = 3.7–5.1) of U.S. older Medicare beneficiaries (or 2.0 million older adults) reported transportation barriers to social/community activities in the preceding month. Compared to 14.1% of those without transportation barriers, all those with transportation barriers were non-drivers, and more than half of them reported that transportation problems kept them from visiting family/friends, attending religious services, attending club meetings, or going out to enjoy. On average, these older adults reported participation in 1.8 of these activities in the past month, compared to 2.6 activities reported by those without transportation barriers. More than one-third of those with transportation barriers reported needing help to go outside, compared to 7.5% of those without transportation barriers. More than a quarter of those with transportation barriers reported that they never/rarely went outside in the past month, compared to 4.2% of those without transportation barriers.

**Table 1.** Characteristics of older adults reporting transportation barriers compared to those not reporting barriers.

Variable	No report of barriers (*n *= 7,052; 95.6%)	Report of barriers (*n *= 495; 4.4%)	*p*
**Transportation barriers and going-out frequency**			
**Driving status (%)**			<.001
** Driving**	85.9	0	
** Not driving**	14.1	100	
**Transportation barriers to (%)**			
** Family/friend visit**	N/A	54.9	
** Religious service attendance**	N/A	52.1	
** Meeting attendance**	N/A	51.9	
** Going out to enjoy**	N/A	55.7	
**No. of social/community activities participated in the past month, mean (*SE*)**	2.58 (0.02)	1.79 (0.06)	<.001
**Types of activities (%)**			
** Family/friend visit**	87.1	64.6	<.001
** Religious service attendance**	52.6	46.3	.030
** Meeting attendance**	39.6	22.2	<.001
** Going out to enjoy**	78.4	45.8	<.001
**Need help going outside the home (%)**	7.5	38.3	<.001
**Frequency of going out in the past month (%)**			<.001
** 5+ days a week**	84.6	42.5	
** 2–3 days a week**	11.3	31.6	
** Rarely/never**	4.2	25.9	
**Sociodemographics, health status, and social support**			
**Age (years, %)**			<.001
** 65–74**	53.1	38.1	
** 75–84**	37.4	32.5	
** 85+**	9.5	29.4	
**Female (%)**	54.1	76.4	<.001
**Race/ethnicity (%)**			<.001
** Non-Hispanic White**	78.7	64.3	
** Non-Hispanic Black**	8.3	14.2	
** Hispanic**	8.1	15.0	
** Other**	4.9	6.5	
**Married/partnered (%)**	58.7	24.6	<.001
**Bachelor’s degree or higher (%)**	36.4	21.8	<.001
**Income <$43,000**	35.8	61.9	<.001
**Residential care community residence (%)**	3.5	14.5	<.001
**No. of chronic medical conditions, mean (*SE*)**	2.32 (0.02)	3.12 (0.10)	<.001
**Past-month activity-limiting pain (%)**	31.0	56.1	<.001
**Dementia (%)**			<.001
** None**	88.0	60.8	
** Possible dementia**	7.0	22.8	
** Probable dementia**	4.9	16.4	
**Social support network size (0–5), mean (*SE*)**	2.25 (0.03)	1.91 (0.07)	<.001
**Video calls with family/friends (%)**	35.7	17.7	<.001

*Note*. *SE* = standard error. All statistics are weighted. Probability values were calculated using Pearson’s χ^2^ tests for categorical variables and *t* tests for continuous variables.

Additional analyses among all non-drivers (14.1% of those not reporting transportation barriers and 100% of those reporting transportation barriers; sample *N *= 2,010; population *N *= 8.3 million older adults) showed that most of them received ride help from family/friends, without a significant difference between those who did (82.6%) and did not (83.6%) report transportation barriers (*F*[1, 56] = 0.115, *p *= .736). Those with and without the report of transportation barriers did not differ on public transportation use, either (16.8% of those reporting barriers vs 15.4% of those not reporting barriers: *F*[1, 56] = 0.286, *p *= .595). However, higher proportions of those who reported barriers than those who did not use van/shuttle services (26.4% vs 16.0%, *F*[1, 56] = 15.262, *p *< .001) and taxi/Uber/Lyft (22.0% vs 14.1%, *F*[1, 56] = 10.504, *p *= .002).

### Sociodemographic and social support differences

Table 1 also shows that, compared to those without, those with transportation barriers were older (9.5% vs 29.4% ages 85+) and included higher proportions of women, racial/ethnic minorities, those with low income (<$43,000), and residential care community residents. Those with transportation problems included lower proportions of married/partnered and college-educated individuals. They also had more chronic medical conditions, more than half reported activity-limiting pain, and 16% (compared to 4.9% of those without transportation problems) had probable dementia. They had a smaller social network size and were less likely to have used video calls to connect with family and/or friends.

### Loneliness and depressive/anxiety symptoms

Table 2 shows that those with transportation barriers experienced loneliness at a significantly higher frequency than those without (*M *= 2.68, *SE *= 0.10 vs *M = *1.81, *SE = 0.01*, *p *< .001). Specifically, compared to 6.0% of those without transportation barriers, 29.2% of those with the barriers reported feelings of loneliness on 5+ days a week. Depressive/anxiety symptoms were also higher among those with transportation barriers (*M *= 3.47, *SE *= 0.20 vs *M *= 1.65, *SE *= 0.04, *p *< .001). Compared to 7.1% of those without transportation barriers, 23.7% of those with the barriers had moderate/severe symptoms.

**Table 2.** Loneliness and depressive/anxiety symptoms.

Variable	No report of barriers (*n = *7,052; 95.6%)	Report of barriers (*n = *495; 4.4%)	*p*
**Loneliness (1 [never] – 5 [everyday]), mean (*SE*)**	1.81 (0.01)	2.68 (0.10)	<.001
**Loneliness by frequency group (%)**			<.001
** Never**	50.0	29.2	
** Rarely (≥ once a week)**	28.0	19.2	
** Some days (2–3 days a week)**	16.0	22.4	
** Most days (5–6 days a week)**	3.1	13.0	
** Every day**	2.9	16.2	
**Depressive/anxiety symptoms (0–12), mean (*SE*)**	1.65 (0.04)	3.47 (0.20)	<.001
**Depressive/anxiety symptoms by severity (%)**			<.001
** None**	74.2	45.6	
** Mild**	18.7	30.7	
** Moderate/severe**	7.1	23.7	

*Note*. *SE* = standard error. All statistics are weighted. Probability values were calculated using Pearson’s χ^2^ tests for categorical variables and *t* tests for the continuous variables.

### Direct effects of transportation barriers and loneliness

Table 3 shows the mediation model results: the direct effects of transportation barriers and loneliness on depressive/anxiety symptoms and the effect of transportation barriers on loneliness. The top rows (direct effects) show that, as hypothesized, depressive/anxiety symptoms were positively associated with transportation barriers (*B *= 0.50, *SE *= 0.19, *t *= 2.70, *p *= .009) and loneliness (*B *= 0.82, *SE *= 0.05, *t *= 16.11, *p *< .001). Depressive/anxiety symptoms were also positively associated with female gender, Hispanic ethnicity, married/partnered state, number of chronic medical conditions, activity-limiting pain, and possible or probable dementia; however, they were negatively associated with ages 85 and older, having a college degree, and video call use with family/friends.

**Table 3.** Effects of transportation barriers and loneliness on depressive/anxiety symptoms.

Model parameter	*B* (*SE*)	*t*	*p*	95% CI
**Depression/anxiety score**				
** Transportation barriers**	0.50 (0.19)	2.70	.009	[0.13, 0.87]
** Loneliness**	0.82 (0.05)	16.11	<.001	[0.72, 0.92]
** Age 75–84**	–0.12 (0.08)	–1.60	.114	[–0.28, 0.03]
** Age 85 and older**	–0.23 (0.10)	–2.26	.028	[–0.43, –0.03]
** Female**	0.30 (0.06)	4.85	<.001	[0.18, 0.42]
** Black**	0.15 (0.09)	1.62	.112	[–0.04, 0.33]
** Hispanic**	0.39 (0.11)	3.61	.001	[0.17, 0.61]
** Other race/ethnicity**	0.21 (0.15)	1.39	.169	[–0.09, 0.50]
** Married/partnered**	0.23 (0.08)	2.89	.005	[0.07, 0.39]
** ≥Bachelor’s degree**	–0.26 (0.05)	–4.77	<.001	[–0.37, –0.15]
** No. of chronic medical conditions**	0.18 (0.03)	6.95	<.001	[0.13, 0.23]
** Activity-limiting pain**	0.98 (0.08)	12.24	<.001	[0.82, 1.15]
** Possible dementia**	0.32 (0.14)	2.27	.027	[0.04, 0.60]
** Probable dementia**	0.82 (0.16)	5.16	<.001	[0.50, 1.13]
** Social support network size**	–0.03 (0.02)	–1.26	.213	[–0.08, 0.02]
** Video calls with family/friends**	–0.22 (0.08)	–2.88	.006	[–0.38, –0.07]
**Loneliness**				
** Transportation barriers**	0.49 (0.10)	4.75	<.001	[0.28, 0.69]
** Age 75–84**	–0.11 (0.03)	–4.05	<.001	[–0.17, –0.06]
** Age 85 and older**	–0.17 (0.04)	–4.24	<.001	[–0.26, –0.09]
** Female**	0.02 (0.03)	0.71	.478	[–0.04, 0.09]
** Black**	–0.19 (0.04)	–4.91	<.001	[–0.27, –0.12]
** Hispanic**	–0.02 (0.05)	–0.47	.641	[–0.11, 0.07]
** Other race/ethnicity**	0.01 (0.09)	0.11	.914	[–0.16, 0.18]
** Married/partnered**	–0.65 (0.04)	–17.43	<.001	[–0.73, –0.58]
** ≥Bachelor’s degree**	–0.01 (0.04)	–0.03	.976	[–0.07, 0.07]
** No. of chronic medical conditions**	0.06 (0.01)	5.10	<.001	[0.23, 0.36]
** Activity-limiting pain**	0.30 (0.03)	9.24	<.001	[0.23, 0.36]
** Possible dementia**	0.14 (0.06)	2.38	.021	[0.02, 0.26]
** Probable dementia**	0.33 (0.06)	5.30	<.001	[0.20, 0.45]
** Social support network size**	–0.02 (0.01)	–1.65	.105	[–0.05, 0.01]
** Video calls with family/friends**	−0.01 (0.03)	–0.29	.772	[–0.08, 0.06]

*Note. N *= 7,537; population size = 46,376,790; number of strata = 56; number of PSUs = 112, Design *df *= 56. The reference group for age 75–84 and age 85 and older was age 65–74; the reference group for Black, Hispanic, and Other race/ethnicity was non-Hispanic White; the reference group for possible and probable dementia was no dementia.

The bottom rows in Table 3 show that, as hypothesized, loneliness was positively associated with transportation barriers (*B *= 0.49, *SE *= 0.10, *t *= 4.75, *p *< .001). Loneliness was also positively associated with the number of chronic medical conditions, activity-limiting pain, and possible and probable dementia; however, it was negatively associated with ages 75–84 and ages 85+, being Black, and a married/partnered state.

### Indirect effects of loneliness on depressive/anxiety symptoms

Bootstrapped results show a significant indirect effect of transportation barriers on depressive/anxiety symptoms through loneliness (0.40, 95% CIs = [0.23, 0.57], *z *= 4.57, *p *< .001). The ratio of the indirect effect of loneliness on transportation barriers (0.40) to the total effect of transportation barriers on depressive/anxiety symptoms (indirect effect [0.40] + direct effect [0.50] = 0.90) was 0.44. The ratio of the indirect effect of loneliness on transportation barriers to the direct effect of transportation barriers on depressive/anxiety symptoms was 0.80. These findings fully support the study hypotheses.

## Discussion

In this study, we examined the extent to which the association between transportation barriers to participating in social/community activities and depressive/anxiety symptoms among older adults is mediated by loneliness. Weighted analyses suggest that about 2 million U.S. older adults, all non-drivers, reported transportation barriers in 2023. Compared to those not reporting transportation barriers, older adults who reported the barriers included higher proportions of individuals in the 85+ age group, women, racial/ethnic minorities, and low-income older adults. These older adults went outside their homes less frequently and participated in fewer social/community activities. Over 80% of those with transportation barriers relied on family/friends for their transportation needs, one in six also used public transportation, a little more than a quarter relied on van/shuttle services, and a little more than one in five used taxi or ride share services.

Consistent with expectations, older adults who reported transportation barriers to social/community activities exhibited a high prevalence of loneliness and depressive/anxiety symptoms, that is, 29% experienced loneliness on five or more days per week, and 24% reported moderate to severe depressive/anxiety symptoms. Our findings highlight a significant indirect pathway: loneliness mediates the association between transportation barriers and depressive/anxiety symptoms. Notably, the ratio of the indirect effect (via loneliness) to the direct effect was 0.80, suggesting that much of the depression/anxiety associated with transportation barriers operates through increased loneliness. When transportation barriers limit access to social engagement, loneliness emerges as a key consequence, contributing to depression/anxiety in later life. These results underscore the critical role of mobility in preventing and reducing loneliness and enhancing mental health among older adults.

As discussed, the link between loneliness and the elevated risks of depression/anxiety has been well established (Cacioppo et al., 2006; Lee et al., 2021; Santini et al., 2020). The experience of chronic loneliness can heighten sensitivity to social threats, increase negative cognitive biases, and reduce perceived social support, all of which are psychological pathways associated with the development of depressive/anxiety symptoms (Cacioppo & Hawkley, 2009). Moreover, loneliness may trigger physiological stress responses, such as cardiovascular stress, inflammation, and dysregulation of the hypothalamic-pituitary-adrenal axis, which have also been implicated in mood and anxiety disorders (Hackett et al., 2012; Steptoe et al., 2004). Thus, loneliness may serve as both a psychological and biological stressor, engendering or exacerbating depressive/anxiety symptoms in later life, especially among those older adults who are socially excluded and isolated.

Our findings show that, as expected, physical and cognitive health problems were associated with both higher loneliness and depressive/anxiety symptoms. However, social support network size was not a significant factor for loneliness and depressive/anxiety symptoms, understandably because a larger network does not necessarily mean that one feels supported or connected, especially among older adults who need instrumental support, such as assistance with transportation. Virtual contact with family/friends was associated with reducing depressive/anxiety symptoms, but it was not associated with reducing loneliness, suggesting that virtual contact may not be enough to prevent or alleviate loneliness. Studies found that increased virtual contact and/or with family/friends during the COVID-19 pandemic was no substitute for decreased in-person contact and was associated with increased loneliness (Choi et al., 2022; Dhakal et al., 2023). These studies showed that physical presence and closeness are important for reducing loneliness.

While individuals aged 75 and older had both lower loneliness and depressive/anxiety symptoms, compared to those aged 65–74, other demographic factors were differently associated with loneliness and depressive/anxiety symptoms. Female gender and Hispanic ethnicity were associated with higher depressive/symptoms but not with loneliness; being Black was associated with lower frequency of loneliness only; and a college degree was associated with lower depressive/anxiety symptoms but not loneliness. A married/partnered state was associated with lower loneliness but higher depressive/anxiety symptoms but with lower loneliness. Being married or partnered may protect against loneliness because there is a built-in social presence of a spouse/partner, which can reduce feelings of social isolation. However, in later life, low quality of the relationship and negative marital interaction are significant factors for increased depression/anxiety (Bulanda et al., 2021; Irani et al., 2022; Nazario-Acevedo et al., 2024). Being in a strained or caregiving-heavy relationship could contribute to stress, conflict, or burden, potentially increasing depressive/anxiety symptoms. These findings of differential moderation effects of demographic and social support variables on loneliness and depression/anxiety indicate that while depression/anxiety, especially among older adults with transportation barriers, is highly correlated with loneliness, there are other factors that separately affect loneliness and depression/anxiety in this population.

These findings align with the social-ecological model, which emphasizes the interplay of structural, community, interpersonal, and individual factors in the link between transportation and health. Transportation barriers at the structural and community levels limit opportunities for social participation, which then manifests at the individual level as loneliness and, ultimately, depressive/anxiety symptoms. The strong indirect pathway observed in our study underscores how upstream mobility challenges cascade into individual mental health outcomes. Interventions, therefore, need to operate across levels, improving affordable and age-friendly transportation systems while also fostering social connections and supportive networks to buffer against loneliness and depression/anxiety. Transportation is not just a mobility issue but a mental health determinant, and our findings have significant implications for fostering mobility and reducing loneliness in later life associated with restricted participation in social/community activities due to transportation barriers.

The overarching policy implications of the findings are that transportation services should be included in broader mental and behavioral health strategies for older adults. Studies have shown that many lonely older adults retain a strong desire to connect with others and contribute to their communities (Smith, 2012; Tomida et al., 2024). Previous studies also found that if and when older adults who stopped driving can maintain their social engagement and use alternative transportation to do so, the psychological effect of driving cessation is mitigated. For instance, a study of Australian older women ages 76–87 who stopped driving found that those who had a strong social support network and remained active in social/community activities were able to maintain a good level of mental health despite driving cessation (Pachana et al., 2016). A study of urban Japanese older adults who stopped driving also found that public transportation users showed a significantly lower level of loneliness than nonusers (Matsuda et al., 2019). These studies indicate that enabling older adults to continue their social engagement and to use alternative transportation means are likely effective strategies for enhancing their mental health outcomes. Given the high proportions of women, racial/ethnic minorities, and low-income older adults among those reporting transportation barriers, reducing transportation barriers could also be an effective way to improve the mental health of those with marginalized social statuses.

Specific policy implications are (1) publicly funded transportation programs (e.g., through the Older Americans Act) should explicitly support social participation as an outcome, not just as part of medical services. (2) Senior centers, community organizations, and transportation providers need to coordinate rides for social, community, and recreational activities, reducing both loneliness and depressive/anxiety symptoms. (3) Public transportation and para-transit services need to be more attuned to the needs and functional abilities of older adults. (4) In areas with limited public transportation, on-demand ride-hailing services can fill critical gaps, especially for non-medical rides (e.g., social/community events) (Bayne et al., 2021; Misra et al., 2022; Mitra et al., 2019). Public–private partnerships between Area Agencies on Aging, community organizations, and ride-hailing companies to subsidize or coordinate rides for older adults need to be expanded. Lonely older adults might especially benefit from ride-hailing services more than their less lonely and more socially connected counterparts (Talmage et al., 2020). To make ride-hailing services older-adult friendly, driver training and accessibility features, for example, assistance with boarding, vehicles that accommodate walkers/wheelchairs, and a telephone-based dispatch system like the GoGo Grandparent program (https://www.gogograndparent.com/), are needed. (5) Social/community activities, not just for older adults but for all age groups, should be located in the neighborhoods where people can walk safely. This will contribute to the community residents’ physical and mental health. Along with the use of alternative transportation, walking or using a wheelchair/scooter to get places was associated with participating in more types of social activities in older adults (Latham-Mintus et al., 2022).

The study’s strength includes a large nationally representative sample, but the study also has some limitations. First, only correlation, not causation, can be derived from cross-sectional survey data. Second, all data were self-reported and may have been subjected to recall and social desirability bias. Third, the analytic models did not include indicators of cultural context, other life-course factors, and the built and social environments.

## Conclusions

Despite these limitations, this study makes a strong case for the importance of transportation in meeting the social needs of older adults, thereby improving their social inclusion and mental health outcomes. Older adults with transportation barriers to participating in social/community activities experience a significantly higher level of loneliness than those without the barriers, which, in turn, is highly associated with greater depressive/anxiety symptoms. The mediating role of loneliness in the association between transportation barriers and depressive/anxiety symptoms is substantial and shows that enabling older adults to continue to engage in social/community activities by alleviating transportation barriers will be the key to preventing and reducing loneliness and depressive/anxiety symptoms in the rapidly growing number of older adults.

## Data Availability

The data used in this study (The National Aging and Health Trends Study) are in the public domain. This study was not preregistered.
